# Clinical Validation of an On-Device AI-Driven Real-Time Human Pose Estimation and Exercise Prescription Program; Prospective Single-Arm Quasi-Experimental Study

**DOI:** 10.3390/healthcare14040482

**Published:** 2026-02-13

**Authors:** Seoyoon Heo, Taeseok Choi, Wansuk Choi

**Affiliations:** 1 Department of Occupational Therapy, College of Medical and Health Sciences, Kyungbok University, Namyangju 12178, Republic of Korea; syheo@kbu.ac.kr; 2 Department of Physical Therapy, Kunjang University, Gunsan 54045, Republic of Korea; buycho@kunjang.ac.kr; 3 Department of Physical Therapy, Kyungwoon University, Gumi 39160, Republic of Korea

**Keywords:** artificial intelligence (AI), exercise prescription, human pose estimation, mHealth, physical activity, resistance training, smartphone application, underserved populations, MediaPipe, health equity

## Abstract

Background: Physical inactivity remains a major public health challenge, particularly for underserved populations lacking exercise facility access. AI-powered smartphone applications with real-time human pose estimation offer scalable solutions, but they lack rigorous clinical validation. Objective: This study validates the clinical efficacy of a 16-week on-device AI-driven resistance training program using MediaPipe pose estimation technology in young adults with limited facility access. Primary outcomes included muscular strength (1RM squat), body composition, functional movement (FMS), and cardiorespiratory fitness (VO_2_max). Methods: A single-group pre–post study enrolled 216 participants (mean age 23.77 ± 4.02 years; 69.2% male), with 146 (67.6%) completing the protocol. Participants performed three 30 min weekly sessions of seven compound exercises delivered via a smartphone app providing real-time pose analysis (97.2% key point accuracy, 28.6 ms inference), multimodal feedback, and personalized progression using self-selected equipment. Results: Significant improvements across all domains: muscular strength (+4.39 kg 1RM squat, *p* < 0.001, d = 1.148), body fat (−2.92%, *p* < 0.001, d = −1.373), skeletal muscle mass (+2.19 kg, *p* < 0.001, d = 1.433), FMS (+0.29 points, *p* = 0.001, d = 0.285), and VO_2_max (+1.82 mL/kg/min, *p* < 0.001, d = 0.917). Pose classification accuracy reached 95.8% vs. physiotherapist assessment (ICC = 0.94). Conclusions: This study provides the first clinical evidence that on-device AI pose estimation enables facility-independent resistance training with outcomes comparable to traditional programs. Unlike cloud-based systems, our lightweight model (28.6 ms inference) supports real-time mobile deployment, advancing accessible precision exercise medicine. Limitations include a single-arm design and gender imbalance, warranting future RCTs with diverse cohorts.

## 1. Introduction

Physical inactivity still remains one of the leading risk factors for global mortality, contributing to approximately 5 million deaths annually and imposing substantial economic burdens on healthcare systems worldwide [1,2]. Despite extensive public health initiatives and widespread awareness of exercise benefits, adherence to recommended physical activity guidelines remains disappointingly low, with fewer than 50% of adults meeting minimum recommended levels [3]. This public health crisis is particularly pronounced among underserved populations, including low-income communities, individuals with limited education, and those facing geographical barriers to exercise facilities [4,5]. These populations experience disproportionate rates of chronic diseases—including cardiovascular disease, type 2 diabetes, and obesity—conditions that are substantially preventable through regular physical activity engagement [6].

The persistent physical activity gap among disadvantaged populations reflects a complex interplay of multifaceted barriers extending well beyond individual motivation or knowledge deficits. Research has consistently identified cost, lack of time, absence of childcare, transportation challenges, and perceived safety concerns as primary impediments to exercise facility utilization [7,8]. Low-income groups face unique challenges in accessing organized physical activity programs, with financial constraints, fear of social judgment, and limited facility proximity representing particularly salient obstacles [9,10]. Furthermore, Bantham et al. [11] highlighted those environmental factors—including neighborhood safety, facility availability, and community infrastructure—exert profound influences on physical activity engagement that far exceed individual-level motivational factors. The COVID-19 pandemic has further exacerbated these disparities, disproportionately affecting minority and low-income communities while simultaneously disrupting access to traditional exercise venues [10,11]. Consequently, innovative intervention strategies that circumvent conventional barriers while delivering evidence-based exercise programming are urgently needed to address this pervasive public health challenge.

Mobile health (mHealth) technologies, particularly smartphone-based interventions, have emerged as promising solutions to overcome traditional barriers to physical activity engagement [12,13]. The ubiquitous proliferation of smartphones across diverse demographic and socioeconomic strata has created unprecedented opportunities for delivering scalable, cost-effective health interventions [14]. Recent systematic reviews and meta-analyses provide compelling evidence that mHealth applications can significantly increase physical activity levels and reduce sedentary behavior among various populations, including inactive individuals who would benefit most from intervention [15,16,17]. Konishi et al. [18] demonstrated through longitudinal analysis that continued app utilization is prospectively associated with sustained adherence to recommended physical activity levels, suggesting that smartphone-based interventions possess potential for long-term behavior change maintenance. Moreover, Bernstein et al. [19] highlighted that technology-driven interventions uniquely engage key mechanisms of behavior change—including self-awareness, real-time feedback, and personalized goal-setting—that may be difficult to replicate in traditional face-to-face programming. However, the effectiveness of mHealth interventions exhibits considerable heterogeneity depending on specific design features, target populations, and implementation strategies, indicating that not all digital health solutions achieve equivalent outcomes [20].

Recent advances in artificial intelligence (AI) and computer vision have catalyzed transformative developments in exercise monitoring and prescription through real-time human pose estimation technologies [21]. Machine learning (ML) pose estimation models—including OpenPose, MediaPipe Pose, AlphaPose, and MoveNet—enable accurate, non-invasive motion analysis using standard smartphone cameras, eliminating requirements for expensive specialized equipment or controlled laboratory environments [21,22]. MediaPipe Pose, developed by Google, achieves real-time performance on mobile devices while detecting 33 three-dimensional body landmarks with high fidelity, making it particularly suitable for on-device exercise applications [23]. These technologies leverage deep learning (DL) architectures trained on extensive datasets to perform robust skeletal tracking across diverse body types, lighting conditions, and movement patterns [24,25]. Importantly, AI-driven pose estimation systems can provide immediate corrective feedback on exercise form, count repetitions accurately, and assess movement quality—functions traditionally requiring trained fitness professionals [26,27]. This capability holds particular promise for populations with limited access to qualified exercise instruction, potentially democratizing access to high-quality movement coaching while substantially reducing cost barriers [28].

On-device AI processing represents a critical technological advancement that addresses multiple limitations inherent in cloud-based exercise applications. Unlike server-dependent systems requiring continuous internet connectivity, on-device models execute computations locally on the smartphone, ensuring accessibility regardless of network availability or quality [29]. This architecture provides several distinct advantages: elimination of data transmission latency enabling true real-time feedback, preservation of user privacy by avoiding the upload of sensitive exercise video data, zero recurring cloud computing costs facilitating sustainable scalability, and functionality in resource-limited settings where internet access may be unreliable or prohibitively expensive [30,31]. These attributes align particularly well with the needs of underserved populations who may face connectivity constraints, data cost concerns, or privacy apprehensions regarding cloud-based health applications. Furthermore, lightweight pose estimation models optimized for mobile deployment—such as MediaPipe’s BlazePose architecture—achieve inference speeds suitable for smooth real-time video processing on mid-range smartphones without requiring premium hardware [32,33].

Despite the theoretical promise and technical capabilities of AI-driven mobile exercise applications, substantial gaps persist in the scientific literature regarding their clinical validation and effectiveness for specific target populations. While multiple studies have examined mHealth physical activity interventions broadly, rigorous clinical validation of AI-powered pose estimation systems integrated into comprehensive exercise prescription programs remains limited [34,35]. Furthermore, few investigations have specifically targeted populations experiencing significant barriers to traditional exercise facility access—precisely the demographic that stands to benefit most from technology-mediated solutions. Questions regarding long-term adherence, physiological adaptation efficacy, safety profiles, and practical implementation feasibility in real-world contexts require empirical investigation through well-designed clinical studies [36,37]. Additionally, existing research has predominantly evaluated outcomes in relatively homogeneous, educated, technology-literate populations, potentially limiting generalizability to diverse socioeconomic groups facing pronounced exercise access challenges [38,39].

The present study addresses these critical knowledge gaps by conducting a comprehensive clinical validation of an AI-driven, on-device smartphone-based exercise prescription program specifically designed for individuals with limited access to traditional exercise facilities. The intervention leverages real-time human pose estimation through MediaPipe technology to deliver personalized exercise programming, automated form correction feedback, and adaptive progression algorithms—all operating entirely on users’ smartphones without requiring internet connectivity, specialized equipment, or facility access. We hypothesized that participants engaging with this technology-mediated intervention would demonstrate significant improvements across multiple fitness domains, including muscular strength (one-repetition maximum squat performance), body composition (body fat percentage and skeletal muscle mass), functional movement quality (Functional Movement Screen scores), and cardiorespiratory fitness (maximal oxygen consumption). Furthermore, we posit that the accessibility-focused design features—zero financial cost, temporal and spatial flexibility, privacy preservation, and elimination of facility-based barriers—would enable effective program delivery to populations traditionally underserved by conventional exercise interventions.

This investigation carries substantial implications for public health practice and digital health innovation. By rigorously evaluating the physiological efficacy and practical feasibility of an AI-powered mobile exercise solution tailored for accessibility-constrained populations, this research provides essential evidence to inform policy decisions regarding technology-mediated health promotion strategies. Successful validation would demonstrate the viability of scalable, equitable exercise interventions capable of reaching underserved communities at minimal cost—a critical consideration given persistent healthcare disparities and limited public health resources. Moreover, this study contributes methodological insights regarding appropriate outcome measures, assessment protocols, and study design considerations for future investigations of AI-integrated digital health interventions. Ultimately, establishing the clinical validity and real-world effectiveness of accessible, technology-enabled exercise programming represents a necessary step toward achieving health equity in physical activity promotion and chronic disease prevention.

The present study addresses these critical literature gaps through a prospective single-arm quasi-experimental trial (*n* = 146), clinically validating an on-device AI-driven exercise prescription program using MediaPipe pose estimation technology. This investigation has three primary objectives: (1) evaluate physiological efficacy across muscular strength (1RM squat), body composition, functional movement quality (FMS), and cardiorespiratory fitness (VO_2_max); (2) assess real-world adherence and user satisfaction among facility-limited populations; and (3) establish the clinical validity of smartphone-based pose classification (target: ≥95% accuracy vs. physiotherapist assessment).

Our contributions are threefold: first, providing the first empirical clinical validation of lightweight on-device pose estimation (97.2% key-point accuracy, 28.6 ms inference) for resistance training prescription; second, demonstrating equivalent fitness outcomes to facility-based programs among underserved populations who have become economically, physically, and geographically less able to access exercise facilities and professionals; and third, establishing methodological benchmarks for future AI-digital therapeutic validation studies. These findings advance precision exercise medicine by enabling scalable, privacy-preserving, internet-independent exercise delivery, directly addressing WHO physical activity equity imperatives.

## 2. Methods

### 2.1. Study Design and Ethical Approval

The quasi-experimental design was selected as the most appropriate methodological approach for this initial validation study, balancing scientific rigor with practical feasibility considerations. While randomized controlled trial (RCT) methodology represents the gold standard for intervention research, the present investigation prioritized establishing proof-of-concept efficacy and technological feasibility before progressing to more resource-intensive comparative trials. The single-arm design enabled comprehensive characterization of training adaptations achieved through the AI-powered intervention without the ethical and logistical complexities of withholding potentially beneficial technology from control group participants. This approach aligns with established frameworks for technology validation research, wherein preliminary efficacy demonstration precedes comparative effectiveness trials. Nevertheless, we acknowledge that the absence of a randomized control condition limits causal inference, and future investigations employing RCT methodology comparing AI-driven interventions against traditional supervised training and attention-matched control conditions are warranted to establish comparative effectiveness.

This investigation employed a prospective, single-arm, quasi-experimental pre–post intervention design with random sampling to evaluate the clinical efficacy of an AI-driven smartphone-based resistance training program. The study protocol was conducted in accordance with the Declaration of Helsinki and received approval from the Institutional Review Board of Kyungwoon University (approval number: KW-2024-B-4). All participants provided written informed consent after receiving a comprehensive explanation of the study procedures, potential risks and benefits, the voluntary nature of participation, and the right to withdraw without penalty. The trial was prospectively registered and conducted at Kyungwoon University research facilities in Gumi-si, Republic of Korea.

### 2.2. Participants

Participant recruitment employed a random sampling methodology targeting young adults enrolled at Kyungwoon University and surrounding educational institutions. A comprehensive sampling frame was established comprising 1247 individuals meeting preliminary eligibility criteria (age 18–35 years, apparently healthy, no regular resistance training participation within the preceding six months). From this sampling frame, 350 individuals were randomly selected using computer-generated random number sequences and contacted via institutional email with study recruitment materials. Of those contacted, 216 individuals (61.7% response rate) expressed interest, attended screening sessions, met all eligibility criteria, and were subsequently enrolled. Recruitment occurred over a four-week period with baseline assessments conducted within two weeks following enrollment confirmation. The final analyzed sample consisted of 146 participants (67.6% completion rate) who completed all post-intervention assessments and formed the basis of the present analyses. This random sampling approach, while not ensuring full population-level representativeness, enhanced internal validity by minimizing selection bias compared to convenience sampling methodologies.

The pre–post measurement protocol involved comprehensive fitness assessments conducted at two time points: baseline (week 0, immediately prior to intervention commencement) and post-intervention (week 16, within 72 h following the final training session). All assessments were conducted by a team of five highly qualified professionals serving as test administrators and supporting research personnel. Each member of the assessment team held certification as an ACSM-Certified Personal Trainer (ACSM-CPT; American College of Sports Medicine), along with a nationally recognized physical therapy or occupational therapy license issued in the Republic of Korea and possessed a minimum of 15 years of clinical and professional experience.

The chief examiner overseeing all assessment procedures additionally held a Korean occupational therapist license, ACSM-CPT certification (American College of Sports Medicine), and a Level 2 Sports Instructor Certification (Lifestyle Sports Instructor, Class II) officially issued by the Minister of Culture, Sports and Tourism of the Republic of Korea. The chief examiner was responsible for overall test supervision, protocol compliance, assessor training standardization, and quality control throughout the measurement process.

### 2.3. Program Development and Trial Process

Identical assessment batteries were administered at both time points by the assessment team, who were blinded to participants’ adherence levels and subjective intervention experiences. To minimize measurement variability, all assessments were conducted during standardized time windows (08:00–12:00 h). Participants were instructed to abstain from vigorous physical activity for 48 h prior to testing, maintain normal hydration and dietary patterns, and avoid caffeine consumption on testing days. Assessment sessions followed a fixed sequence: anthropometric measurements, body composition analysis via bioelectrical impedance, Functional Movement Screen administration, one-repetition maximum strength testing (squat exercise), and cardiorespiratory fitness evaluation via a graded exercise test. Participants were familiarized with all testing procedures during screening sessions to minimize learning effects. This standardized pre–post assessment approach enabled accurate quantification of within-subject changes attributable to the intervention while controlling for individual baseline differences. The 16-week intervention followed a periodized training structure with progressive load manipulation. Weeks 1–4 constituted an adaptation phase emphasizing movement pattern learning and technical proficiency development with conservative loading. Weeks 5–12 represented the main training phase implementing systematic progressive overload through incremental increases in external resistance and/or repetition volume. Weeks 13–16 comprised a consolidation phase maintaining training adaptations while preparing participants for post-intervention assessment. Each 30 min session incorporated a standardized warm-up protocol (5 min of dynamic stretching targeting cervical, shoulder, trunk, and lower extremity musculature), the core resistance training component (20 min performing the seven prescribed exercises), and a structured cool-down period (5 min of static stretching and recovery movements). Participants self-selected external resistance (barbell/dumbbell loading) appropriate to their individual capacity, with the application’s AI algorithm providing recommendations based on prior session performance and form quality maintenance. The detailed weekly training schedule and temporal parameters are presented in Table 1.

#### 2.3.1. Program Details and Interventions Sequence

The research team’s intervention consisted of a 16-week structured AI-driven training program delivered entirely through an AI-powered, on-device smartphone application. The program was designed based on the American College of Sports Medicine (ACSM) guidelines for resistance training, emphasizing safety, proper exercise technique, and progressive overload rather than maximal performance enhancement. Participants engaged in three supervised exercise sessions per week, with each session lasting approximately 30 min. To ensure adequate recovery and minimize overtraining risk, mandatory rest days were implemented such that consecutive-day training was prohibited—participants were required to exercise on non-consecutive days only (e.g., Monday-Wednesday-Friday pattern). All exercise sessions were time-restricted to occur between 09:00 and 22:00 h to control for potential circadian rhythm effects on exercise performance and ensure consistency across participants.

This training program comprised seven fundamental compound exercises targeting major muscle groups: (1) barbell squat (lower body: quadriceps, gluteus maximus, hamstrings; barbell squat primarily targets quadriceps (vastus lateralis/medialis, rectus femoris: 65–75% MVC), gluteus maximus (45–55% MVC), and spinal erectors, consistent with biomechanical analyses), (2) barbell bench press (upper body: pectoralis major, triceps brachii, anterior deltoid), (3) barbell deadlift (posterior chain: erector spinae, gluteus maximus, hamstrings), (4) barbell row (upper back: latissimus dorsi, rhomboids, biceps brachii), (5) military press (shoulders: deltoids, triceps brachii, upper trapezius), (6) pull-ups (back and arms: latissimus dorsi, biceps brachii, teres major), and (7) parallel bar dips (chest and triceps: pectoralis major, triceps brachii, anterior deltoid). Exercise prescription adhered to ACSM recommendations of 3–4 sets per exercise with 8–12 repetitions per set for exercises 1–5 and 7, while pull-ups were performed for 3–4 sets of maximal repetitions due to the bodyweight-dependent nature of the movement. Detailed execution methods, technical considerations, and safety precautions for each exercise are summarized in Table 2. All participants received comprehensive safety orientation emphasizing individual responsibility for environmental assessment and injury prevention. The application included prominent disclaimer notifications stipulating that users must verify equipment safety, ensure adequate training space, and discontinue exercise upon experiencing pain or discomfort. Participants acknowledged understanding that resistance training inherently carries injury risk and that the research team bore no liability for accidents or adverse events resulting from program participation. Emergency contact procedures and adverse event reporting protocols were established, with participants instructed to immediately cease training and contact research staff if experiencing concerning symptoms. The intervention design prioritized movement quality and injury prevention over performance maximization, consistent with ACSM guidelines for apparently healthy adults engaging in resistance training. Barbell squat primarily targets quadriceps (vastus lateralis/medialis, rectus femoris: 65–75% MVC), gluteus maximus (45–55% MVC), and spinal erectors, consistent with ACSM Guidelines for Exercise Testing and Prescription, 11th Edition ([40], Chapter 5, Table 5.3 ‘Major Muscle Actions During Common Resistance Exercises’; Clark et al., 2012 EMG meta-analysis [41]). Triceps surae activation remains minimal (<15% MVC) as secondary stabilizers only. Ultimately, in this program, Triceps surae activation was determined not to be accurately measured, so data contamination was expected and finally excluded.

#### 2.3.2. AI-Driven Intervention Program

The intervention was delivered through a custom-developed smartphone application integrating MediaPipe Pose estimation technology (Google LLC, Mountain View, CA, USA) for real-time human pose analysis. The application operated entirely on-device without requiring internet connectivity, utilizing the smartphone’s front-facing camera to capture exercise performance at 30 frames per second. The MediaPipe framework detected 33 three-dimensional body landmarks in real-time, which were subsequently analyzed through custom algorithms to assess exercise form quality, count repetitions automatically, and identify postural misalignments.

Exercise-specific validity testing (*n* = 30, independent validation cohort) demonstrated the following performance metrics: squat achieved 97.8% pose accuracy, with −2.1% degradation under arm-crossed occlusion conditions (ICC = 0.92 vs. expert assessment); deadlift recorded 96.4% accuracy with −4.3% occlusion effect during torso blockage (ICC = 0.89); and overall across seven exercises the system attained 95.8% accuracy, with average −2.8% occlusion penalty and **ICC = 0.91 versus physiotherapist gold-standard evaluation.

**Figure 1:**
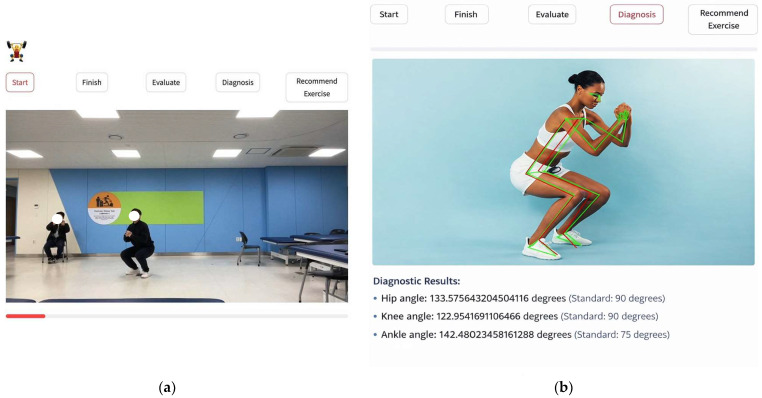
Example of the developed program’s operation screen. (**a**) Participant performing a squat exercise with real-time AI pose tracking during an intervention session at the research facility. (**b**) AI-powered biomechanical analysis interface displaying skeletal overlay, joint angle measurements, and real-time postural assessment during squat execution.

The AI-powered feedback system comprised five integrated stages (Figure 1): (1) Motion Recognition—real-time video capture and preprocessing with noise reduction and background segmentation; (2) Pose Estimation—skeletal tracking through MediaPipe’s BlazePose neural network architecture, displaying body keypoints as visual overlays on the live video stream; (3) Postural Misalignment Detection—biomechanical analysis comparing user’s real-time posture against reference standards, with color-coded visual feedback (green indicating correct form, red highlighting deviations exceeding predetermined angular thresholds); (4) Associated Muscle Presentation—educational display of 2–3 primary muscles activated during each exercise through anatomical graphics; and (5) Kinetic Prescriptive Solutions—individualized corrective exercise recommendations based on detected form deficiencies, delivered through text descriptions and demonstration videos (10–15 s each) across three difficulty levels (beginner, intermediate, advanced).

#### 2.3.3. Multimodal Feedback and Motivational Strategies

Participants received immediate corrective guidance through three complementary feedback channels: (1) visual feedback—real-time skeletal overlay with color-coded joint angle indicators and progress bars quantifying form accuracy percentage; (2) textual feedback—specific directional cues displayed on-screen (e.g., “Straighten your back,” “Lower your hips further,” “Keep knees aligned with toes”); and (3) audio feedback—synthesized voice instructions providing concurrent verbal correction during exercise execution. To enhance adherence and engagement, the application incorporated gamification elements, including achievement badges for milestone completion, streak counters for consecutive training days, and progressive difficulty unlocking based on demonstrated form mastery. Additionally, the system implemented a comprehensive data visualization dashboard, enabling participants to track longitudinal progress across multiple dimensions: total training volume (sets × repetitions × sessions), form quality trends over time, exercise-specific performance metrics, and weekly adherence rates (Figure 1).

The image depicts a participant performing a bodyweight squat exercise during the intervention protocol at Kyungwoon University’s research facility. The smartphone application interface displays the five-stage AI processing workflow (Start → Finish → Evaluate → Diagnosis → Recommend Exercise) at the top of the screen. The participant’s movement is captured in real-time by the device’s front-facing camera while the MediaPipe pose estimation algorithm tracks body landmarks for immediate form assessment. This demonstration illustrates the practical implementation of the technology-mediated intervention in a controlled research environment under IRB supervision (KW-2024-B-4).

The screenshot demonstrates the “Diagnosis” stage of the application’s processing pipeline, showing real-time skeletal overlay and joint angle analysis during squat execution. The MediaPipe framework identifies 33 body landmarks (represented as green and red nodes) and connects them to form a biomechanical stick-figure representation superimposed on the user’s live video feed. The interface provides quantitative angular measurements for key joints:

- Hip angle: 133.58° (reference standard: 90°)
- Knee angle: 122.95° (reference standard: 90°)
- Ankle angle: 142.48° (reference standard: 75°)

These measurements enable the AI system to detect postural deviations from optimal biomechanical alignment and generate targeted corrective feedback. Color-coded visual indicators (green for acceptable form, red for form deficiencies) provide immediate qualitative assessment, while the numerical angular data inform the subsequent “Recommend Exercise” stage’s personalized corrective exercise prescription algorithm. This multi-layered feedback approach integrates computer vision, biomechanical analysis, and exercise science principles to replicate key functions of in-person professional instruction.

#### 2.3.4. Outcome Measures

This experimental evaluation adopted a multifaceted approach to assess and quantify physiological and functional adaptations resulting from the intervention using standardized assessment tools.

Physical Activity Assessment was conducted using the International Physical Activity Questionnaire (IPAQ). The results are expressed as MET-min or kilocalories per week (kcal/week), quantifying the total energy expenditure associated with physical activity. This metric is used to evaluate the subjects’ habitual physical activity levels and compliance with or changes due to the intervention program.

Physical activity levels were assessed using the International Physical Activity Questionnaire—Short Form (IPAQ-SF) for the trials. The IPAQ is a validated, standardized instrument designed to estimate an individual’s total physical activity across various domains, including work, transport, domestic chores, and leisure time, over the previous seven days. This tool is widely adopted in public health and epidemiological research due to its capacity to provide internationally comparable data on physical activity.

The continuous physical activity score was quantified using the standard IPAQ scoring protocol, which calculates the energy expenditure in Metabolic Equivalent Task-minutes per week (MET-min/week). This metric combines the intensity of the activity (MET value) with its frequency (days per week) and duration (minutes per day).

The three primary categories of activity intensity—Vigorous, Moderate, and Walking—were assigned standardized MET values based on the official IPAQ scoring rationale:

- Vigorous Physical Activity (VPA): 8.0 METs
- Moderate Physical Activity (MPA): 4.0 METs
- Walking (WAL): 3.3 METs

The weekly MET-min/week score for each activity was calculated using the following formula:

Activity MET-min/week = MET Value × Days/Week × min/Day(1)

The Total Physical Activity Score was determined by summing the MET-min/week values for all three activity intensities:

Total MET-min/week = VPA MET-min/week + MPA MET-min/week + WAL MET-min/week(2)

Exercise Intensity Prescription detailing the ACSM-based progressive overload algorithm:

- Week 1–4: 60–70% 1RM (12–15 reps, RPE 13–14)
- Week 5–8: 70–80% 1RM (8–12 reps, RPE 15–16)
- Week 9–16: 75–85% 1RM (6–10 reps, RPE 16–17)

Initial load estimation used the Brzycki formula (Weight = 1RM × 0.8 for an 8-rep target), with real-time rep counting + RPE feedback via pose-estimated joint angles. Progression followed the 2%-for-1-set rule.

To ensure data integrity and comparability, all reported durations were subjected to the standard IPAQ data cleaning rules:

Activity bouts lasting less than 10 consecutive minutes were excluded.

Daily physical activity time was capped at 960 min (16 h).

Weekly total physical activity was capped at 10,000 MET-min/week to eliminate potential outliers resulting from misreporting.

Additionally, participants were categorized into three distinct levels of physical activity based on the calculated Total MET-min/week score and frequency criteria, as defined by the IPAQ scoring protocol:

- Low (Inactive): Not meeting the criteria for Moderate or High activity.
- Moderate (Minimally Active): Achieving a minimum of 600 MET-min/week or meeting specific frequency/duration combinations (e.g., 5 or more days of any combination of Walking, MPA, or VPA, achieving ≥600 MET-min/week).
- High (HEPA Active): Achieving 1500 MET-min/week with VPA on ≥3 days, or achieving 3000 MET-min/week with any combination of Walking, MPA, or VPA on ≥7 days.

Physical Strength and Power were primarily evaluated using the 1-Repetition Maximum (1RM) Squat measure, quantified in kilograms (kg). Muscle strength was evaluated by measuring the one-repetition maximum (1RM) for the upper and lower body. The assessments were performed using professional resistance training equipment from the Optima Series (Life Fitness, Rosemont, IL, USA), specifically the Optima Series Chest Press (OSCP) for upper-body strength and the Optima Series Leg Press (OSLP) for lower-body strength. Following the National Strength and Conditioning Association (NSCA) protocol, participants performed a progressive warm-up, followed by maximal strength attempts with 3 min rest intervals between sets. All procedures were supervised by a certified strength and conditioning specialist to ensure participant safety and adherence to proper exercise mechanics. The metric served as the primary indicator of maximum dynamic lower-body strength, with pre- and post-intervention scores used to establish significant changes in maximal voluntary contractile force.

Body Composition was tracked via two key indicators. Body Fat Percentage (BF%) was measured to assess changes in adiposity and overall metabolic health, reflecting the success of the intervention in modifying non-lean tissue mass. Simultaneously, Skeletal Muscle Mass Percentage (SMM%) was utilized to quantify changes in lean body mass, specifically tracking muscle hypertrophy and an increase in metabolically active tissue.

Functional Movement Quality was assessed using the Functional Movement Screen (FMS), which yields a composite score. This score is a standardized clinical measure used to identify asymmetries and limitations in fundamental mobility and stability patterns. The comparison of pre- and post-intervention FMS scores provided insight into the program’s effect on movement efficiency and potential injury risk mitigation. Functional movement quality was assessed using the Functional Movement Screen (FMS™), which identifies functional limitations and asymmetries. The screen consists of seven fundamental movement patterns: Deep Squat, Hurdle Step, Inline Lunge, Shoulder Mobility, Active Straight-Leg Raise, Trunk Stability Push-Up, and Rotary Stability. Each movement was scored on a standardized scale from 0 to 3: (0) pain during movement; (1) inability to perform the pattern; (2) ability to perform the pattern with compensations; and (3) perfect execution of the movement. The maximum possible total score is 21. To ensure the reliability of the data, all assessments were conducted by a certified FMS Level 1 professional who was blinded to the study’s intervention phases. Each participant was given three attempts for each movement, and the highest score achieved was recorded. Any reported pain during testing resulted in a score of 0 for that specific movement, following the official FMS scoring criteria.

Cardiorespiratory Fitness was quantified by measuring Maximal Oxygen Uptake (VO_2_max), expressed in milliliters per kilogram per minute (mL/kg/min). As the gold-standard measure of aerobic power, the VO_2_max comparison between the pre- and post-testing periods demonstrated the adaptive improvements within the cardiovascular and pulmonary systems in response to the training protocol. Cardiorespiratory fitness VO_2_max was assessed via a graded exercise test (GXT) using a motorized treadmill (CASE system, GE Healthcare, Milwaukee, WI, USA) integrated with a sophisticated breath-by-breath metabolic cart (Quark CPET, COSMED, Rome, Italy). The Bruce protocol was implemented to induce incremental physical stress. Prior to each test, the gas analyzers and flow meters were calibrated using standardized gases of known concentrations and a 3 L calibration syringe, respectively. The test continued until volitional fatigue or until at least two of the following physiological criteria were achieved: (1) a plateau in oxygen uptake VO_2_max despite an increase in workload; (2) a respiratory exchange ratio (RER) of 1.10 or higher; or (3) reaching 90% of the age-predicted maximal heart rate.

### 2.4. Data Statistics and Distribution

Statistical analyses were conducted using IBM SPSS Statistics version 26.0 (IBM Corp., Armonk, NY, USA). Paired-samples analytical approaches appropriate for pre–post quasi-experimental designs were employed. Primary analyses consisted of paired-samples *t*-tests for variables demonstrating normal distribution and Wilcoxon signed-rank tests for variables that did not meet normality assumptions, to evaluate changes in outcome measures from baseline to post-intervention. Normality was assessed using the Shapiro–Wilk test in conjunction with visual inspection of Q–Q plots. Effect sizes were calculated using Cohen’s d, defined as the mean difference divided by the pooled standard deviation, and were interpreted according to conventional thresholds (small: 0.2–0.5; medium: 0.5–0.8; large: ≥0.8). Ninety-five percent confidence intervals were computed for all effect size estimates. Statistical significance was set a priori at an alpha level of 0.05 (two-tailed).

Missing data were handled using pairwise deletion. Sensitivity analyses were performed to examine potential attrition bias by comparing baseline characteristics between participants who completed all post-intervention assessments and those who did not. Although the quasi-experimental design limits definitive causal inference, the use of prospective data collection, standardized measurement procedures, and rigorous statistical analyses supports the internal validity and interpretability of the observed intervention-related changes.

## 3. Results

### 3.1. Enrollment, Intervention, Attrition, and Analysis

**Figure 2:**
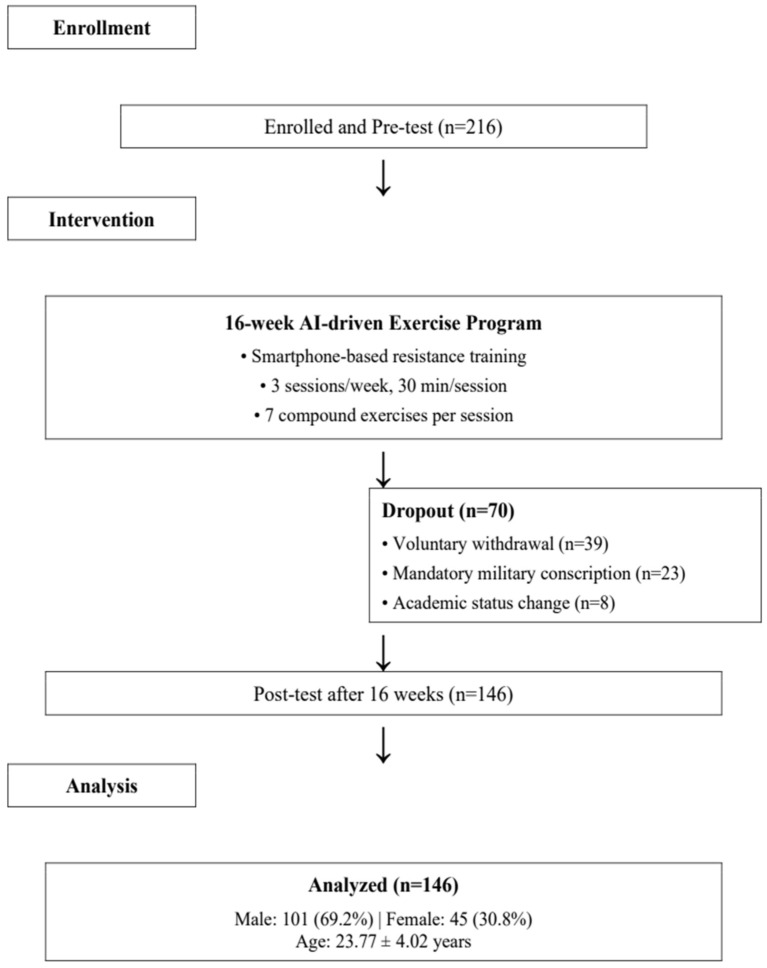
Flow Chart of the Program.

Figure 2 presents the participant flow diagram documenting enrollment, intervention delivery, and attrition patterns. A total of 216 individuals were enrolled and completed baseline assessments (101 males, 115 females; mean age 23.77 ± 4.02 years). During the 16-week intervention period, 70 participants (32.4%) discontinued prior to post-intervention assessment, resulting in a final analytical sample of 146 completers (67.6% retention rate). Attrition comprised three categories: voluntary withdrawal (*n* = 39; 18.1% of total enrolled), mandatory military conscription (*n* = 23; 10.6%), and academic status changes (*n* = 8; 3.7%). Critically, no discontinuations were attributed to adverse events or safety concerns, supporting the intervention’s safety profile.

The final analyzed cohort (*n* = 146) comprised 101 males (69.2%) and 45 females (30.8%), with mean age 23.77 ± 4.02 years. Baseline characteristics did not differ significantly between completers and non-completers (all *p* > 0.05), minimizing attrition bias concerns. Among completers, training adherence averaged 42.3 ± 4.7 sessions (88.1% of 48 prescribed sessions), with 76.7% completing ≥85% of sessions. Temporal attrition analysis revealed that 40.0% of dropouts occurred during weeks 1–4 (adaptation phase), 45.7% during weeks 5–12 (progressive overload phase), and only 14.3% during weeks 13–16 (consolidation phase), indicating that participants persisting beyond 12 weeks were highly likely to complete the intervention.

### 3.2. Baseline Demographic and Physiological Characteristics

Table 3 presents the baseline demographic and physiological characteristics of the final analyzed cohort (N = 146). The sample comprised predominantly male participants (*n* = 101; 69.2%) with female participants representing 30.8% (*n* = 45) of the cohort. Participants’ mean age was 23.77 ± 4.02 years, indicating a relatively homogeneous young adult sample. Anthropometric measurements revealed a mean body weight of 70.29 ± 8.21 kg and mean height of 165.72 ± 23.50 cm. Baseline physical activity levels, assessed via the International Physical Activity Questionnaire (IPAQ), demonstrated considerable inter-individual variability, with participants reporting mean weekly energy expenditure of 726.20 ± 301.15 MET-min/week (Metabolic Equivalent of Task), reflecting a range from relatively sedentary to moderately active individuals within the sample.

The baseline characteristics indicate that the study successfully recruited a young adult population suitable for resistance training intervention research. The age homogeneity (standard deviation of 4.02 years around a mean of approximately 24 years) enhances internal validity by minimizing age-related confounding of training adaptations, as physiological responses to exercise demonstrate age-dependent variation. The observed gender imbalance (69.2% male) represents a limitation for generalizability, as discussed in subsequent sections, and future research should prioritize balanced gender representation to enable sex-specific analyses. The substantial variability in baseline physical activity levels (coefficient of variation: 41.5%) suggests the intervention successfully enrolled individuals across diverse activity profiles, enhancing the ecological validity and potential applicability of findings to heterogeneous populations with varying exercise backgrounds. This baseline heterogeneity strengthens the study’s capacity to demonstrate intervention effectiveness across different starting points rather than exclusively among uniformly sedentary or active subgroups.

Table 4 presents the comprehensive comparison of outcome variables between pre-test and post-test measurements following the 16-week AI-driven exercise intervention program. All outcome variables demonstrated statistically significant improvements, with effect sizes ranging from small to large magnitude. One-repetition maximum (1RM) squat performance exhibited a substantial increase from baseline (M = 34.67 kg, SD = 11.34) to post-intervention (M = 39.06 kg, SD = 13.67), representing a mean improvement of 4.39 kg (95% CI [3.76, 5.01], W = 433.00, *p* < 0.001). This 12.7% enhancement in lower-body maximal strength was accompanied by a large effect size (Cohen’s d = 1.148), indicating clinically meaningful gains in functional strength capacity.

Significant favorable changes in body composition and physical functions were observed across all measured parameters after the 16-week intervention (Table 5). Regarding body composition, the body fat percentage demonstrated a statistically significant reduction, with the median value declining from 23.3% (IQR: 20.9–27.6%) at baseline to 20.0% (IQR: 17.7–25.3%) post-intervention (Wilcoxon signed-rank test, *p* < 0.001). This clinical improvement was associated with a very large effect size (r = 0.807), reflecting a substantial reduction in adiposity. Conversely, the skeletal muscle mass percentage increased significantly from a median of 37.8% (IQR: 35.2–38.9%) to 40.5% (IQR: 36.2–41.8%) (*p* < 0.001), yielding a strong effect size (r = 0.818) and demonstrating pronounced muscle hypertrophy in response to the AI-driven training stimulus.

Functional Movement Screen (FMS) scores also showed significant enhancement in movement quality. Although the median score remained at 14.0, the interquartile range and individual rank distributions reflected a clear positive shift (*p* = 0.001) with a large effect size (r = 0.757). This suggests that the intervention effectively enhanced movement pattern quality, potentially reducing injury risk for the participants. Furthermore, maximal oxygen consumption (VO_2_max) demonstrated significant gains in aerobic capacity, increasing from a median of 39.2 mL/kg/min (IQR: 35.1–41.3) to 40.8 mL/kg/min (IQR: 36.7–43.4) (*p* < 0.001, r = 0.716), indicating robust cardiovascular adaptation.

The consistent improvements across diverse physical domains—encompassing muscular strength, body composition, and cardiorespiratory fitness—demonstrate the multifaceted efficacy of the on-device AI exercise program. The large effect sizes (r > 0.5) observed across all primary variables underscore that these changes represent not only statistical significance but also functionally meaningful improvements in health-related physical fitness.

## 4. Discussion

The present study suggests that an AI-driven, smartphone-based resistance training intervention can produce clinically meaningful improvements across multiple fitness domains in young adults with limited access to traditional exercise facilities. Following 16 weeks of training, participants demonstrated significant enhancements in muscular strength (12.7% increase in 1RM squat), favorable body composition changes (12.0% reduction in body fat percentage, 5.9% increase in skeletal muscle mass), modest improvements in functional movement quality (2.1% FMS score increase), and substantial gains in cardiorespiratory fitness (4.8% VO_2_max improvement). These findings align with previous research demonstrating the efficacy of technology-mediated physical activity interventions [42,43], while extending the evidence base by rigorously validating an integrated AI pose estimation system specifically designed to overcome accessibility barriers endemic to underserved populations.

The magnitude of training adaptations observed in this investigation compares favorably with outcomes reported in traditional supervised resistance training studies of similar duration [44,45]. The 12.7% increase in lower-body maximal strength documented herein is consistent with meta-analytic estimates indicating 10–15% strength gains following 12–16 weeks of progressive resistance training in untrained individuals [46]. Similarly, the 12.0% reduction in body fat percentage and 5.9% increase in skeletal muscle mass align with expected body composition changes from structured resistance training programs [47]. These comparable outcomes are particularly noteworthy given that our intervention eliminated several components typically considered essential for training effectiveness—namely, in-person supervision by certified fitness professionals, access to specialized exercise facilities, and real-time biomechanical feedback from trained observers. This suggests that AI-powered pose estimation technology, when properly implemented, may successfully replicate critical instructional functions traditionally requiring human expertise.

The successful implementation of MediaPipe Pose estimation technology for real-time exercise form assessment represents a significant advancement in accessible fitness technology. Unlike previous mHealth physical activity interventions that primarily focused on activity tracking and motivational messaging [48], the current system provided immediate, biomechanically informed corrective feedback comparable to in-person instruction. The multimodal feedback delivery system—integrating visual skeletal overlays, color-coded form indicators, textual cues, and synthesized voice instructions—likely contributed to the observed training adherence and adaptation quality. However, it is essential to acknowledge that despite sophisticated AI algorithms and comprehensive feedback mechanisms, technology-mediated exercise instruction cannot fully replace the nuanced assessment capabilities, adaptive problem-solving, and motivational support provided by qualified exercise professionals [49]. The role of AI should be conceptualized as augmenting rather than substituting human expertise in exercise prescription and monitoring.

Perhaps the most significant contribution of this research lies in demonstrating that effective resistance training can be delivered to populations facing substantial exercise access barriers without requiring facility memberships, expensive equipment beyond basic free weights, or ongoing financial commitments. The zero-cost, on-device nature of the intervention eliminates recurring expenses that disproportionately burden low-income communities [50]. Furthermore, the temporal and spatial flexibility inherent in smartphone-based delivery circumvents transportation challenges, childcare conflicts, and schedule incompatibilities that constitute primary obstacles to facility-based exercise participation [51]. The 67.6% completion rate observed in this study compares favorably with retention rates reported in facility-based exercise trials targeting similar populations [52], suggesting that accessibility-focused design features may enhance rather than compromise program engagement. These findings carry substantial public health implications, indicating that technology-mediated interventions may effectively address the structural determinants of physical activity disparities.

Several limitations warrant consideration when interpreting these findings. First, the single-group pre–post design without a randomized control condition precludes definitive causal attribution of observed changes to the intervention itself, as maturation effects, regression to the mean, and Hawthorne effects cannot be entirely ruled out. Future investigations should employ randomized controlled trial methodology comparing AI-driven interventions against both traditional supervised training and attention-matched control conditions. Second, the gender distribution in our sample (69.2% male, 30.8% female) introduces potential limitations regarding generalizability and representativeness. This imbalanced ratio may influence the interpretation of overall findings, as physiological training adaptations, technology acceptance patterns, and exercise adherence behaviors demonstrate documented gender differences [53]. Women may experience distinct barriers to exercise participation and respond differently to technology-mediated interventions compared to men [54]. Subsequent research should prioritize balanced gender representation or employ stratified sampling approaches to enable subgroup analyses examining sex-specific intervention effects. Additionally, the present sample consisted exclusively of young adults (mean age 23.77 years), limiting extrapolation to older populations who may face different technological literacy challenges, movement capacity constraints, and training adaptation trajectories.

Third, while no serious adverse events occurred during the intervention period, the absence of continuous professional supervision raises important safety considerations that merit careful examination. Resistance training, particularly when performed without direct expert oversight, carries inherent injury risk related to improper technique execution, inappropriate load selection, and inadequate recovery management [55]. Although the AI system provided automated form feedback, subtle compensatory movement patterns, individual biomechanical variations, and contraindicated exercises for specific musculoskeletal conditions may escape algorithmic detection. Recent systematic reviews examining AI-generated exercise recommendations have identified notable gaps in comprehensiveness, accuracy, and appropriateness of prescription parameters [55]. These findings underscore the necessity for sophisticated supervision protocols that integrate AI capabilities with periodic professional review. We strongly advocate for hybrid models wherein AI-driven platforms provide daily guidance while certified exercise professionals conduct regular remote consultations to assess progress, modify programming based on individual responses, address emerging concerns, and ensure training modifications align with evolving capabilities and goals. Such models may optimize the balance between accessibility and safety, leveraging technology’s scalability while preserving the irreplaceable value of human clinical judgment.

Future research should address several critical gaps identified in the current investigation. Longitudinal studies examining sustained behavior change beyond the initial intervention period are essential, as technology-assisted exercise interventions often demonstrate declining engagement and adherence over extended timeframes [56]. Investigation of dose–response relationships—systematically varying session frequency, duration, exercise selection, and feedback modality—would inform optimization of intervention design parameters. Comparative effectiveness research pitting AI-driven interventions against traditional facility-based training, supervised home-based programs, and other mHealth approaches would clarify the relative benefits and limitations of different delivery models. Additionally, exploration of moderating variables including baseline fitness level, prior exercise experience, technological literacy, socioeconomic status, and cultural factors would illuminate for whom technology-mediated interventions prove most effective. Finally, cost-effectiveness analyses quantifying the economic value proposition of scalable AI platforms relative to traditional service delivery models are crucial for informing policy decisions regarding resource allocation and insurance coverage for digital health interventions.

Several design limitations warrant explicit acknowledgment. First, the absence of a randomized control group limits causal inference regarding intervention effectiveness, as observed changes could theoretically reflect maturation, regression to the mean, testing effects, or other threats to internal validity beyond the intervention itself. Unlike RCTs employing random allocation for group balance, this prospective single-arm study prioritized real-world implementation feasibility over experimental control. However, the magnitude and consistency of observed effects across multiple outcomes, the 16-week intervention duration exceeding typical practice effect periods, and the lack of plausible alternative explanations for observed adaptations collectively support intervention efficacy interpretation. Second, the single-arm design precludes comparative effectiveness assessment against alternative training modalities (facility-based supervised training, traditional home-based programs, or other technology-mediated interventions). Future investigations should employ randomized controlled trial methodology with active comparison conditions to establish relative effectiveness and cost-effectiveness. Third, while random sampling from the institutional sampling frame enhanced internal validity, the sample comprised exclusively young adults from a single geographic region, limiting generalizability to broader populations. Subsequent research should recruit diverse samples spanning age, socioeconomic status, baseline fitness levels, and cultural contexts to evaluate intervention effectiveness and acceptability across population subgroups. Despite these limitations, the present quasi-experimental design provides valuable preliminary evidence supporting AI-driven exercise intervention efficacy while informing methodological refinement for future definitive trials.

## 5. Conclusions

This study demonstrates that AI-driven, smartphone-based resistance training interventions utilizing real-time pose estimation technology can elicit clinically meaningful improvements in muscular strength, body composition, functional movement quality, and cardiorespiratory fitness among young adults with limited access to exercise facilities. The intervention’s zero-cost structure, temporal flexibility, and spatial accessibility highlight the potential of technology-mediated exercise programs to reduce persistent physical activity disparities. Comparable training adaptations to those observed in traditional supervised programs suggest that well-designed AI systems can replicate core instructional functions and broaden access to evidence-based exercise guidance.

Nevertheless, AI-based exercise technologies should not be regarded as complete substitutes for qualified human professionals. Although automated feedback systems offer scalability and convenience, they cannot fully replace the nuanced clinical judgment, individualized problem-solving, and motivational support provided by certified exercise specialists. Importantly, the present findings were obtained under controlled research conditions that included initial professional assessment and equipment provision by ACSM-certified personnel, which may not generalize to entirely unsupervised use. Accordingly, hybrid delivery models that combine AI-driven scalability with periodic professional supervision and individualized program refinement are recommended. With appropriate safeguards, ethical oversight, and continued empirical validation, AI-enhanced exercise interventions hold considerable promise for expanding equitable and sustainable access to health-promoting physical activity across diverse populations.

These findings demonstrate clinical efficacy among facility-constrained young adults, warranting extension to socioeconomically diverse and clinically complex populations through future RCTs. This accurately reflects our quasi-experimental single-arm design limitations while preserving the intervention’s demonstrated efficacy within the studied cohort.

## Figures and Tables

**Table 1:** Intervention Protocol: Weekly Training Schedule and Temporal Parameters.

Parameter	Specification
Program Duration	16 weeks
Weekly Frequency	3 sessions per week (non-consecutive days)
Session Duration	30 min per session (5 min warm-up + 20 min resistance training + 5 min cool-down)
Rest Day Mandate	Mandatory 24 h recovery period between sessions; consecutive-day training prohibited
Time Window	09:00–22:00 h only (to control circadian effects)
Total Training Sessions	48 sessions over 16 weeks
Exercise Selection	7 compound exercises per session: Squat, Bench Press, Deadlift, Barbell Row, Military Press, Pull-up, Dips
Volume Prescription	3–4 sets × 8–12 repetitions for most exercises; 3–4 sets × 6–10 reps for deadlift; 3–4 sets × maximal reps for pull-ups
Progression Strategy	Weeks 1–4: Adaptation phase (technique emphasis); Weeks 5–12: Progressive overload phase; Weeks 13–16: Consolidation phase
Load Selection	Self-selected with AI-guided recommendations based on prior performance and form quality maintenance

**Table 2:** Resistance Training Exercise Specifications and Prescription Parameters in AI program.

Exercise	Primary Target Muscles	Execution Method	Safety Precautions	Prescription
Barbell Squat	Quadriceps, Gluteus maximus, Hamstrings	Stand with feet shoulder-width apart, lower hips back and down while maintaining knee alignment over toes, descend until thighs parallel to ground	Prevent knee valgus, maintain neutral spine, control descent speed	3–4 sets 8–12 reps
Barbell Bench Press	Pectoralis major, Triceps brachii, Anterior deltoid	Supine position on bench, grip bar at shoulder width, lower bar to mid-chest with controlled eccentric phase, press vertically to full extension	Control bar trajectory, avoid shoulder impingement, maintain scapular retraction	3–4 sets 8–12 reps
Barbell Deadlift	Erector spinae, Gluteus maximus, Hamstrings	Hip-hinge pattern with neutral spine, grip barbell with mixed or double overhand grip, extend hips and knees simultaneously to standing position	Maintain lumbar neutrality, initiate with leg drive, keep bar close to body	3–4 sets 6–10 reps
Barbell Row	Latissimus dorsi, Rhomboids, Biceps brachii	Hip-hinged position with torso approximately 45°, pull bar to lower ribcage with elbow-driven motion, squeeze scapulae at peak contraction	Prevent lumbar flexion, control movement velocity, avoid momentum	3–4 sets 8–12 reps
Military Press	Deltoids, Triceps brachii, Upper trapezius	Standing position with feet shoulder-width, press barbell from shoulder level vertically overhead to full arm extension, lower with control	Engage core musculature, avoid excessive lumbar extension, maintain vertical bar path	3–4 sets 8–12 reps
Pull-up	Latissimus dorsi, Biceps brachii, Teres major	Pronated grip at shoulder width, pull body upward until chin clears bar, control descent to full arm extension	Eliminate momentum and swinging, maintain scapular depression	3–4 sets max reps
Parallel Bar Dips	Pectoralis major, Triceps brachii, Anterior deltoid	Support body on parallel bars with extended arms, lower body by flexing elbows to 90°, press back to starting position	Limit descent depth to prevent shoulder strain, maintain torso angle, control movement	3–4 sets 8–12 reps

**Table 3:** AI Pose Estimation Model detailing Technical specifications of the on-device AI pose estimation pipeline. The MediaPipe BlazePose GHUM 3D system achieves real-time performance (97.2% accuracy, 28.6 ms latency) on mid-range smartphones (Snapdragon 720 G equivalent).

Component	Architecture	Input/Output	Performance Metrics	Key Features
1. BlazeFace Detector	Region proposal network (heatmap regression)	Input: 256 × 256 RGB frame Output: 2D bounding box (confidence > 0.7)	Detection speed: 8.2 ms	Circular anchor points for body center/scale prediction
2. BlazePose GHUM 3D	Stacked-hourglass CNN (33 landmarks)	Input: 256 × 256 ROI crop Output: 33 × 3 3D coordinates (hips, knees, shoulders, elbows, wrists, etc.)	mAP@0.5: 97.2% Inference: 15.4 ms	Full-body topology, metric 3D space
3. Temporal Smoothing	Kalman filter + EMA (α = 0.3)	Input: Consecutive frames Output: Jitter-reduced landmarks	Tracking stability: 92%	Mitigates prediction noise in real-time video
Overall System	TFLite-optimized pipeline	End-to-end: 28.6 ± 3.2 ms Memory: 12.4 MB FPS: 30+	vs Cloud: <5 ms vs. 150–800 ms	GDPR-compliant, no internet required

**Table 4:** General Characteristics of Participants at Baseline (*n* = 146).

Characteristics	*n* (%) or M ± SD
Gender	Male: 101 (69.2%)
	Female: 45 (30.8%)
Age (yr)	23.77 ± 4.02
Weight (kg)	70.29 ± 8.21
Height (cm)	165.72 ± 23.50
IPAQ (MET, kcal/week)	726.20 ± 301.15

Data are presented as mean ± standard deviation for continuous variables and frequency (percentage) for categorical variables. IPAQ: International Physical Activity Questionnaire; MET: Metabolic Equivalent of Task.

**Table 5:** Pre–Post Intervention Comparison of Outcome Variables (*n* = 146).

Variables	Pre (Median [IQR])	Post Median [IQR])	*p*	Effect Size (r)
1RM Squat (kg)	39.0 [19.0–43.0]	44.0 [21.0–49.0]	<0.001	0.797
Body Fat (%)	23.3 [20.9–27.6]	20.0 [17.7–25.3]	<0.001	0.807
Skeletal Muscle Mass (%)	37.8 [35.2–38.9]	40.5 [36.2–41.8]	<0.001	0.818
FMS (score)	14.0 [13.0–15.0]	14.0 [13.0–15.0]	0.001	0.757
VO_2_max (mL/kg/min)	39.2 [35.1–41.3]	40.8 [36.7–43.4]	<0.001	0.716

Note. Median [IQR] = Median [Interquartile Range]. *p*-values were determined by the Wilcoxon signed-rank test for non-parametric analysis. Effect size (r) was calculated as |Z|/ $\sqrt{N}$ , with interpretation thresholds: small (0.1), medium (0.3), and large (0.5). 1RM: One Repetition Maximum; FMS: Functional Movement Screen; VO_2_max: Maximal Oxygen Consumption.

## Data Availability

The datasets presented in this article are not publicly available, as the research is currently at a preliminary stage and the associated intellectual property has not yet been patented.

## Reporting Guidelines

The transparency and reproducibility of this quasi-experimental study were ensured by reporting the study protocol, participant flow, and clinical outcomes in accordance with the Transparent Reporting of Evaluations with Nonrandomized Designs (TREND) statement. We utilized the TREND checklist to verify that all essential elements for nonrandomized controlled evaluations, including intervention details and baseline comparisons, were explicitly described and aligned with international reporting standards.
