# Correction: Heo et al. Clinical Validation of an On-Device AI-Driven Real-Time Human Pose Estimation and Exercise Prescription Program; Prospective Single-Arm Quasi-Experimental Study. *Healthcare* 2026, *14*, 482

**DOI:** 10.3390/healthcare14091116

**Published:** 2026-04-22

**Authors:** Seoyoon Heo, Taeseok Choi, Wansuk Choi

**Affiliations:** 1 Department of Occupational Therapy, College of Medical and Health Sciences, Kyungbok University, Namyangju 12178, Republic of Korea; syheo@kbu.ac.kr; 2 Department of Physical Therapy, Kunjang University, Gunsan 54045, Republic of Korea; buycho@kunjang.ac.kr; 3 Department of Physical Therapy, Kyungwoon University, Gumi 39160, Republic of Korea

## Funding Update

There was an error in the funding statement of the original publication [1]. The funder “The Institutional Research Grant Program of Kyungwoon University (Kyungwoon University: 2410010037) (Grant Year: 2025)” was included in the main text mistakenly. The correct funding statement appears below:

**Funding:** This research was supported by the Regional Innovation System & Education (RISE) program through the Gyeongbuk Rise Center, funded by the Ministry of Education (MOE) and the Gyeongsangbuk-do, Republic of Korea (2025-RISE-15-102).

The authors state that the scientific conclusions are unaffected. This correction was approved by the Academic Editor. The original publication has also been updated.
